# Meeting human resources for health staffing goals by 2018: a quantitative analysis of policy options in Zambia

**DOI:** 10.1186/1478-4491-8-15

**Published:** 2010-06-30

**Authors:** Aaron Tjoa, Margaret Kapihya, Miriam Libetwa, Kate Schroder, Callie Scott, Joanne Lee, Elizabeth McCarthy

**Affiliations:** 1 Clinton Health Access Initiative, Boston, USA; 2 The Ministry of Health, The Government of the Republic of Zambia, Lusaka, Zambia; 3 Clinton Health Access Initiative, Lusaka, Zambia; 4 Harvard School of Public Health, Boston, Massachusetts, USA

## Abstract

**Background:**

The Ministry of Health (MOH) in Zambia is currently operating with fewer than half of the health workers required to deliver basic health services. The MOH has developed a human resources for health (HRH) strategic plan to address the crisis through improved training, hiring, and retention. However, the projected success of each strategy or combination of strategies is unclear.

**Methods:**

We developed a model to forecast the size of the public sector health workforce in Zambia over the next ten years to identify a combination of interventions that would expand the workforce to meet staffing targets. The key forecasting variables are training enrolment, graduation rates, public sector entry rates for graduates, and attrition of workforce staff. We model, using Excel (Office, Microsoft; 2007), the effects of changes in these variables on the projected number of doctors, clinical officers, nurses and midwives in the public sector workforce in 2018.

**Results:**

With no changes to current training, hiring, and attrition conditions, the total number of doctors, clinical officers, nurses, and midwives will increase from 44% to 59% of the minimum necessary staff by 2018. No combination of changes in staff retention, graduation rates, and public sector entry rates of graduates by 2010, without including training expansion, is sufficient to meet staffing targets by 2018 for any cadre except midwives. Training enrolment needs to increase by a factor of between three and thirteen for doctors, three and four for clinical officers, two and three for nurses, and one and two for midwives by 2010 to reach staffing targets by 2018. Necessary enrolment increases can be held to a minimum if the rates of retention, graduation, and public sector entry increase to 100% by 2010, but will need to increase if these rates remain at 2008 levels.

**Conclusions:**

Meeting the minimum need for health workers in Zambia this decade will require an increase in health training school enrolment. Supplemental interventions targeting attrition, graduation and public sector entry rates can help close the gap. HRH modelling can help MOH policy makers determine the relative priority and level of investment needed to expand Zambia's workforce to target staffing levels.

## Background

The human resources for health (HRH) shortage is estimated at more than 4 million workers globally [1]. The shortage of health workers is particularly acute in resource-limited settings where it limits the provision of even basic health services like antenatal care and infant immunizations, and it prevents progress towards the health-related Millennium Development Goals (MDG) of improved maternal and child health and universal access to HIV/AIDS treatment [1-4].

Several factors contribute to this shortage of health workers. In some countries, underinvestment in training institutions has led to an inadequate supply of professional health graduates [5-7]. Meanwhile, many qualified health professionals migrate abroad to fill more lucrative health positions [7-11]. Others join the private health sector or leave the health sector altogether [3,12,13].

Policies to reduce HRH shortages include expanding training institutions and providing incentives to improve retention [14,15]. Such policies are being written into national multi-year, ministry-level HRH strategic plans [16]. However, deciding among them or determining the most appropriate level of investments in them presents a significant challenge for decision-makers, as there is uncertainty around predicting the effects of interventions over time or the interplay between them [17].

### HRH Shortage in Zambia

The Republic of Zambia is among the countries currently facing an acute HRH shortage. According to the Government of the Republic of Zambia Ministry of Health (MOH), the country is operating with fewer than half the health workforce necessary to deliver basic health services, with even higher vacancy rates in rural areas [18]. Staff-to-population ratios nationally are as low as 1 doctor per 14 500 people and 1 nurse per 1800 people [19,20]; this is much lower than the 1 health worker per 400 people recommended by the Joint Learning Initiative as the minimum threshold necessary to provide equitable coverage of basic health services [1]. This shortage of health workers is threatening adequate and equitable health care delivery, and it is one of the major factors holding back attainment of the Millennium Development Goals [21].

In 2005, the MOH initiated a policy reform process to address this critical shortage in the public sector [21]. The Zambian MOH established employment targets for each health worker cadre based on recommended World Health Organization (WHO) staff-to-population ratios (2.3 to 1000) with modifications by the MOH and health facility managers [7]. The Zambian Human Resources for Health Strategic Plan 2006 to 2010 established targets and strategies to achieve them through improvements in HRH management, training, and retention [21]. However, to establish the best combination of strategies, a modelling exercise was required. Our objective was to provide practical guidance on this question by estimating the training, hiring, and retention combinations that would enable the MOH to reach its targets in the next ten years.

Other modelling efforts have been developed to establish optimal HRH staffing levels for planning purposes. As reviewed elsewhere, models are often needs-based, utilization-based, service-target based, adjusted-service-to-target, or health workforce-to-population-based estimates [22]. Models are useful planning tools and allow decision-makers to anticipate the impact of certain decisions under different scenarios. For example, scaling up HIV services in Mozambique and the associated health worker requirements were modelled to assist workforce planning [23]. Here we present single variable and multiple variable scenario analyses of the supply of health workers in a model that uses health workforce to population ratios to understand minimum staffing requirements in Zambia.

## Methods

### Study design

We built an HRH projection model to estimate the size of the government health workforce in 2018. We focused our analysis on four key cadres: doctors, clinical officers, nurses, and midwives. These cadres account for 80% of current clinical staff and 75% of employment targets. We forecasted HRH supply under current conditions and then estimated the effect on the size of the government health workforce by modelling changes in training enrolment, graduation rates, public sector entry rates of graduates, and attrition rates.

### HRH projection model

Our HRH projection model uses Excel (Microsoft Office, Microsoft; 2007) to forecast the annual number of health workers in the public sector workforce for each cadre based on the annual inflows and outflows of each cadre in the public sector health workforce. The annual number of staff leaving the workforce includes the number of health workers going back to school as well as those lost to attrition. Annual attrition is calculated by measuring the size of each cadre before new hires multiplied by the workforce attrition rates for each cadre. The annual inflow of staff is equal to the sum of new hires from training institutions and from abroad. To calculate the number of hires from training institutions in a year, the model multiplies together the number of training institution students in their final year of study from the prior year, the graduation rate for the prior year, and the rate that graduates entered the public sector workforce in the previous year:

To avoid overproduction of health workers, the model does not allow the number of health workers in the public sector to exceed the target number for each cadre. If a cadre is expected to reach its target level, the model reduces the number of training enrolment slots in advance of that point so the workforce will meet but not exceed the target number.

The model allows the user to establish attrition rates, immigration inflow, training enrolment, graduation rates, and public sector entry rates for each year. We assume that training institution enrolment slots can always be filled with qualified students.

### Model parameters

#### Workforce parameters

Baseline estimates for key staff (doctors, clinical officers, nurses, and midwives) come from Ministry of Health payroll data from September 2008 (Table 1) [24,25]. The model categorizes attrition as voluntary or involuntary. Voluntary attrition occurs when a health worker who is still able to perform her duties in the public sector workforce chooses to exit the workforce permanently such as leaving the health sector. Involuntary attrition occurs when a health worker leaves permanently due to a condition that would prevent her from continuing to work in the public sector workforce (e.g. serious illness, dismissal, death, or retirement). We assume 68% of total attrition is involuntary and 32% is voluntary based on a Ministry of Health survey [26]. The components of involuntary attrition in the survey include dismissal (12%), retirement (10%), contract expiration or transfer (8%), and serious illness and death (38%)--with AIDS being the leading cause of death. Voluntary resignation (32%) is the only component of voluntary attrition [26].

**Table 1:** Baseline inputs

			Nurses		Midwives			
				
Baseline inputs	Doctors	Clinical officers	registered	enrolled	registered	enrolled	direct entry	Total
Number of staff								

At baseline (2008)^1^	806	1236	1453	5134	339	1711	0	10 679

Minimum level required to meet need (same for all years)^2^	1778	3737	14 053		4751			24 319

Inflow from training								

Annual training enrolment (# of new students)^3^	74	155	660	423	136	173	174	1795

Total years of training required^3^	7	3	3	2	1	1	2	n/a

Graduation rate (% of students)^3^	90.0%	90.0%	96.7%	94.3%	97.1%	90.7%	90.0%	n/a

Public sector entry rate from training schools (% of graduates)^3,4^	85.3%	91.4%	75.5%	81.4%	81.4%	81.4%	81.4%	n/a

Inflow from immigration (# per year)^4^	20	0	0	0	0	0	0	20

Outflow from attrition								

Training program requires other professional diploma/degree and work experience before enrolling; or enrols students who leave the public sector workforce before enrolling	No	No	Yes	No	Yes	Yes	no	n/a

If yes, which specific cadre is the feeder cadre for training programs	n/a	n/a	enrolled nurses	n/a	registered nurses	enrolled nurses	n/a	n/a

Percent of enrolling students to which entry barrier applies (% per year)	n/a	n/a	14.89%	n/a	100%	100%	n/a	n/a

Total attrition from cadre (% per year)	9.80%	4.48%	5.30%	4.48%	4.48%	4.48%	4.48%	n/a

*Involuntary attrition (% per year) *[24,26]	6.66%	3.05%	3.60%	3.05%	3.05%	3.05%	3.05%	n/a

*Voluntary attrition (% per year) *[25,26]	3.14%	1.43%	1.70%	1.43%	1.43%	1.43%	1.43%	n/a

^1 ^MOH human resources payroll data, September 2008

^2 ^MOH Training and Development Plan 2008

^3 ^MOH/CHAI training institution assessment, May/June 2008

^4 ^MOH Human Resources Directorate

#### Training parameters

Training parameter estimates were derived from a 2008 joint Ministry of Health and Clinton Foundation assessment of all 39 medical training institutions in Zambia. The assessment determined graduation rates to be 90-97%, based on available data and interviews with school staff [27].

The same assessment determined that enrolled and registered midwifery training programs require all their students to have nursing professional health diplomas and prior work experience. Additionally, it found 15% of students in the registered nursing program to be former enrolled nurses who left the workforce for further schooling. We use this information to determine the number of nurses who leave the workforce annually to go back to school.

#### Hiring parameters

For simplicity, we assumed graduates are hired into the public sector and begin working within one calendar year after their graduation year for all cadres. To estimate the proportion of new graduates who enter the public sector, we divided the aggregate number of new hires during the period of January 2007 to February 2008 by the number of graduates from training institutions during the same time period [28]. In Zambia, all new non-doctor hires come from Zambian training institutions, and all new doctor hires come from either Zambian training institutions or other countries in the region, with 20 new doctors hired from abroad annually [28].

#### What-if analyses

We conducted what-if analyses to estimate the effects of changes in training, hiring, and attrition conditions on the supply of HRH over time. We assumed all changes would take effect by 2010.

## Results

### Base case analysis

Under current conditions, the number of doctors, clinical officers, nurses, and midwives in the public sector is expected to increase from 10 679 in 2008 to 14 402 in 2018, or from 44% to 59% of minimum necessary staff--far short of the 24 319 recommended by the government (Table 2). The increase in these key healthcare workers from 2008 to 2018 is expected to be a result of an influx of 14 030 new hires that enter into the public sector and a loss of 10 307 existing staff who exit. The sources of the new hires are 13 830 graduates from training institutions and 200 doctors from abroad.

**Table 2:** Projected changes in Zambian HRH workforce from 2008 to 2018 under current conditions of production and attrition

	Doctors	Clinical officers	Nurses	Midwives	Total
Projected HRH workforce in 2018 ( = baseline + inflow - outflow)	792	1828	7508	4274	14 402

Baseline HRH workforce	806	1236	6587	2050	10 679

Inflow from 2008 to 2018 ( = a - b - c + d)	+ 768	+ 1275	+ 8359	+ 3628	+ 14 030

a Students that enrolled	+ 740	+ 1550	+ 10 831	+ 4830	+ 17 951

b Students that failed to graduate	- 74	- 155	- 463	- 375	- 1067

c Graduates not hired into public sector	- 98	- 120	- 2009	- 827	- 3054

d Hired from abroad	+ 200	+ 0	+ 0	+ 0	+ 200

Outflow from 2008 to 2018 ( = e + f + g + h)	- 782	- 683	- 7438	- 1404	- 10 307

e Retire	- 78	- 68	- 337	- 140	- 623

f Involuntary attrition	- 454	- 396	- 1951	- 815	- 3616

g Voluntary attrition	- 250	- 219	- 1077	- 449	- 1995

h Go back to school	- 0	- 0	- 4073	- 0	- 4073

Total gain or loss between 2008 to 2018	- 14	+ 592	+ 921	+ 2224	+ 3723

Remaining staffing gap in 2018	986	1909	6545	477	9917

This table contains outputs from the HRH projection model.

Notably, 4121 students are not expected to enter the public workforce either because of their failure to graduate (1067) or because they graduate but will seek employment elsewhere (3054) (Table 2). Of the 10 307 key health workers who leave the workforce during the 10 year period, 4073 (40%) leave to go back to school, 4239 (41%) leave for involuntary reasons, and 1995 (19%) leave for voluntary reasons.

By cadre, the number of doctors is expected to decrease by 14 with no changes in current trends, while the number of clinical officers, nurses, and midwives are expected to increase by 592, 921, and 2224 respectively over the ten year period. Shortfalls from the minimum requirement remain significant by 2018 without changes in current trends (986 for doctors, 1909 for clinical officers, 6545 for nurses and 477 for midwives).

### Single variable what-if analyses

We projected the number of key healthcare workers in the public sector under several single-variable (one-at-a-time) intervention scenarios that would take effect by 2010. These included increasing the graduation rate to 100%, increasing the public workforce entry rate to 100%, decreasing voluntary attrition to 0%, doubling training enrolment, and tripling training enrolment (Table 3). By itself, with no changes in attrition and hiring rates from current trends, increasing training enrolment had the largest impact on the size of the total workforce by 2018.

**Table 3:** Projected impact of single interventions on the HRH workforce from 2008 to 2018

Single intervention scenario	Number of health workers in 2018 (% of target level)				
	
	Combined	Doctors	Clinical officers	Nurses	Midwives
Baseline projection (no changes)	14 402 (59.2%)	792 (44.5%)	1828 (48.9%)	7508 (53.4%)	4274 (90.0%)

Increase graduation rate to 100% by 2010	15 049 (61.9%)	826 (46.5%)	1920 (51.4%)	7831 (55.7%)	4472 (94.1%)

Decrease voluntary attrition to 0% by 2010	16 199 (66.6%)	958 (53. 9%)	2000 (53.5%)	8713 (62.0%)	4528 (95.3%)

Increase public workforce entry rate to 100% by 2010	16 619 (68.3%)	846 (47.6%)	1906 (51.0%)	9144 (65.1%)	4723 (99.4%)

Double health training institution enrolment by 2010	19 108 (78.6%)	874 (49.2%)	2462 (65.9%)	11 021 (78.4%)	4751 (100%)

Triple health training institution enrolment by 2010	22 669 (93.2%)	957 (53.8%)	3095 (82.8%)	13 866 (98.7%)	4751 (100%)

However, each type of intervention has a different effect on each cadre. For example, decreasing voluntary attrition to 0% has the same impact by 2018 on the number of doctors--who have the highest annual voluntary attrition rate at 3.14%--as tripling training enrolment. Increases in training enrolment have the largest effects for clinical officers, nurses, and midwives who each require only up to three years to train, compared to doctors who require 7 years to train in Zambia.

Under the single-variable intervention scenarios, increases in training enrolment were the only interventions with enough potential power to reach public sector staffing targets by 2018. To reach the combined cadre target of 24 319 staff by 2018, training enrolment must grow by a factor of thirteen for medical doctors (from 74 to 960 per year), quadruple for clinical officers (from 155 to 623 per year), triple for nurses (from 1083 to 2924 per year), and grow by a quarter for midwives by 2010 (from 483 to 589 per year). However, in the scenario of only increasing training enrolment, the Government of Zambia could significantly reduce training enrolment from these higher new enrolment levels after it reaches its staffing targets. Once a scale-up of the workforce to staffing targets is completed by 2018 under the training enrolment scenario, the number of new hires only has to equal the number of new exits from the workforce. The equilibrium level of training enrolment necessary to sustain the workforce at the target level once it has been reached is 201 for medical doctors (172% increase over baseline), 204 for clinical officers (32% increase over baseline), 275 for nurses (18% increase over baseline), and 285 for midwives (41% decrease of baseline), assuming no changes to the current hiring and attrition rates.

### Multi-variable what-if analyses

**Figure 1:**
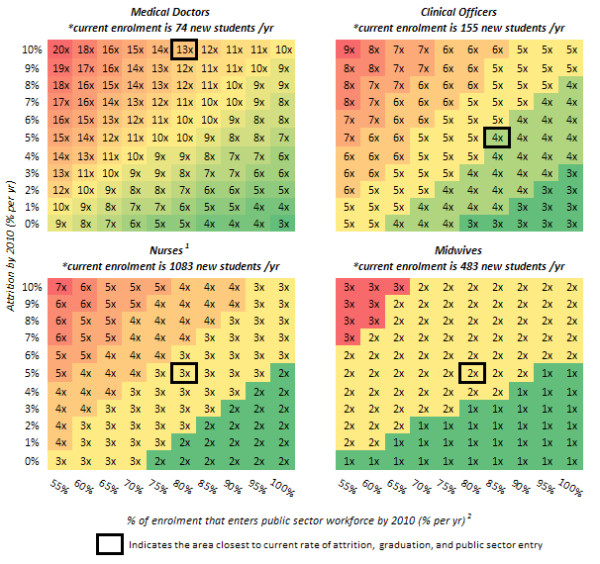
**Minimum changes in training enrolment, attrition, graduation, and public sector entry by 2010 that will achieve staffing targets for each cadre by 2018**. *the increase in training enrolment is described as a multiplier of current training enrolment, i.e. 1× implies no change in enrolment, 2× implies a doubling of enrolment or 100% increase in enrolment, etc; please note that these multipliers were rounded up to the nearest integer. ^1 ^This assumes that the number of midwives trained each year remains at 2008 levels through 2018. ^2 ^This axis is the graduation rate multiplied by the public sector entry rate. This figure highlights the factor by which current training enrolment must increase by 2010 in order to reach staffing targets by 2018, for each change in attrition, graduation, and public sector entry that is achieved by 2010. The x-axis represents the percent enrolment into the public sector from training institutions (graduation rate multiplied by the public sector entry rate) and the y-axis represents percent attrition. A 1× means that training enrolment remains at current levels, while a 2× signifies the need for a doubling of current training enrolment, and so on. A bold box indicates the current rate of attrition, graduation, and public sector entry and the corresponding necessary increase in training enrolment if these other variables remained constant. For example, if 80% of graduating doctors entered the public sector, and attrition were 10% (both of which are close to current rates), then training enrolment would need to increase 13-fold in order to produce enough doctors to meet the targets set for 2018. If there were an increase in graduation and public sector entry and a reduction in attrition, the factor by which training enrolment would need to increase could be brought down as low as three.

We conducted a four-way what-if analysis to determine which combinations of changes in training enrolment, the graduation rate, the public sector entry rate, and attrition would enable the MOH to reach its staffing targets by 2018. The analysis determined that staffing targets could be reached by 2018 for each rate of attrition, graduation, and public sector entry rate achieved by 2010, as long as there is a large enough accompanying increase in training enrolment by 2010 (figure 1).

Doctor targets could be reached by 2018 with smaller increases in training enrolment by reducing attrition and increasing the graduation and public sector entry rate of doctors by 2010. Without changes in current levels of attrition, graduation and public sector entry rates, training enrolment of doctors would have to be increased by a factor of thirteen (13×). However, if attrition is reduced to 0% and graduation and public sector entry is increased to 100% by 2010, the increase in training enrolment required falls to a factor of three (3×) for doctors. As a mid-range scenario, decreasing attrition by five percentage points for doctors could change the necessary training enrolment increase from thirteen times to ten times current levels. Alternatively, the same benefits could be achieved by increasing the combined graduation and public sector entry rate by twenty percentage points.

Reducing attrition and increasing graduation and public sector entry rate by 2010 for clinical officers, nurses, and midwives does not have as significant an effect on the required increase in the size of the training enrolment increase as it does for doctors. At current levels of attrition, training enrolment must be increased by a factor of four (4×) for clinical officers, three (3×) for nurses, and two (2×) for midwives by 2010 in order to reach targets by 2018. If attrition is reduced to 0% and graduation and public sector entry is increased to 100% by 2010, the increase in training enrolment required only falls to a factor of three (3×) for clinical officers, two (2×) for nurses, and one (1×) for midwives. In the case of midwives, however, reducing attrition by two percentage points or increasing graduation and public sector entry by fifteen percentage points from current levels by 2010 will enable the MOH to meet midwives staffing targets by 2018 without an increase in training enrolment (1×).

If attrition rates were to increase or the graduation and public sector entry rate were to fall over time, it would make reaching doctor targets by 2018 even more difficult. For every increase in the attrition rate by two percentage points or for every drop in the graduation and public sector entry rate by ten percentage points, current doctor training enrolment would have to increase by an additional one hundred to two hundred percentage points to reach staffing targets by 2018. The level by which clinical officer and nurse training enrolment must increase to reach staffing goals by 2018 is also sensitive to increases in the attrition rate or decreases in the graduation and public sector entry rate, but much less so than it is for doctors. Roughly, for every increase in the attrition rate by four percentage points or for every decrease in the graduation and public sector entry rate by fifteen percentage points, there would have to be a one hundred percentage point increase in training enrolment to reach staffing targets by 2018 for both clinical officers and nurses. Increases in attrition or decreases in the graduation and public sector entry rate do not significantly affect the training enrolment needs for midwives to reach staffing goals by 2018.

## Discussion

We projected the supply of HRH in the public sector in Zambia from 2008 to 2018 under a number of scenarios. Zambia will not reach target levels for health workers in the next ten years at current levels of training institution enrolment, attrition, and graduation and public sector entry rates, thereby threatening the country's ability to provide adequate and equitable health care delivery to meet its MDGs.

Our analysis identified several optimal combinations of changes in training institution enrolment, attrition, graduation rates, and public sector entry rates to enable Zambia to employ its required number of health workers for the public sector by 2018. In any scenario, a significant increase in training institution enrolment is critical for all cadres but midwives; doubling, tripling or even expanding by up to 13 times the current levels of training enrolment is required for doctors, clinical officers, and nurses.

Doctors require the largest increase in training enrolment to meet minimum staffing needs within the decade because they have the longest training time. Clinical officers and nurses require significant but much smaller increases in training enrolment (by a factor of four and three respectively). Targets for midwives can be met with either an increase in training enrolment or an intervention that improves retention, graduation, and public sector entry rates. Interventions that improve retention, graduation, and public sector entry rates are not sufficient on their own to reach staffing targets by 2018 for doctors, clinical officers, or nurses, but they can reduce the size of the required increase in training enrolment, especially for doctors.

Increasing training enrolment is the most expensive option. It takes several years for training enrolment changes to have an impact on the public workforce. This delay is equal to the length of the training and hiring pipeline for each cadre- the time that it takes for students to be trained, graduate, and enter the workforce. By cadre, the pipeline is eight years for medical doctors, four years for clinical officers, three years for enrolled nurses, four years for registered nurses, two years for registered midwives, two years for enrolled midwives, and three years for direct entry midwives. To increase the workforce in the near future, training enrolment would need to increase immediately. Nonetheless, policies to increase training levels will not address immediate staffing needs, particularly for cadres with the longest pipelines (medical doctors and clinical officers).

Policy options could address the duration of the pipelines. Fast-tracked training programs could produce staff more quickly, or new cadres could be created that require less time to train. Decreasing the amount of time that it takes for the MOH to recruit and hire graduates could also reduce the overall pipeline by up to a year.

Our model also identified another opportunity to reduce immediate shortages--by reducing the number of nurses who leave the workforce to go back to school to get advanced training. Removing prerequisites to advanced nursing degrees (by allowing direct entry) would reduce back-to-school attrition. Zambia has introduced two such programs already: the direct-entry midwifery diploma and the direct-entry nursing bachelor's degree. The direct-entry midwifery diploma does not require students to have a nursing diploma, and it only requires two years of training compared to one year of training for the registered and enrolled midwifery diplomas. The non-direct entry nursing bachelor's degree requires students to have a nursing diploma (minimum two years training) and two years of nursing experience prior to enrolment, but the direct-entry program requires neither. The direct-entry degree only requires three years of training compared to two years of training for a non-direct entry nursing bachelors.

Nurses who leave the workforce to earn higher degrees will not all re-enter the public sector workforce after graduating, as they may choose more lucrative jobs in the private sector or abroad where demand for health workers is high. The estimated rate of retaining the nurses who leave the public sector to go back to school is equal to the graduation rate multiplied by public sector entry rate of each program, currently around 73-79%.

The sizes of the training scale-up necessary to reach staffing targets for doctors, clinical officers, and nurses by 2018 are likely unfeasible or prohibitively costly. Therefore, even with significant gains in improving training enrolment, retention, graduation, and public sector entry, Zambia is likely to operate with a shortage of manpower for at least the next decade unless alternative means of addressing this shortage are determined. For example, shifting some clinical tasks from doctors or clinical officers to nurses and midwives could leverage the ability of nurses and midwives to care for significantly more patients in the short run. Clerical and simple clinical work (e.g. measuring a patient's height, weight, and vitals) could potentially be performed by lay health workers. Nurse- and clinical officer-centred care have resulted in good clinical outcomes in Zambia, where these two cadres provided the bulk of paediatric HIV care in a number of primary health care clinics in Lusaka [29]. Similar results have been found elsewhere, for example in Rwanda where nurses have provided paediatric antiretroviral therapy and in Kenya with family planning services [30,31]. Rationally redistributing tasks among health worker teams (task-shifting), according to a recent review, can maintain quality while increasing efficiency and improving access and affordability [32]. In a pilot in Rwanda, the demand for physician time in providing HIV care and treatment was reduce by 76% by expanding the role of nurses in HIV services [33]. If Zambia were able to replicate these results, the expansion of doctor training enrolment, while still necessary, would not have to be as extreme.

If Zambia increases training enrolment significantly, it is unclear what Zambia would do with a large excess of training graduates once current staffing needs are met. The Philippines has experienced significant economic benefits from the remittances of nurses who emigrate to developed countries, though such a policy would have to be carried out carefully in Zambia so as to avoid the cannibalization of staff hires and the potential for increased outflows from the domestic public sector health workforce due to emigration [34]. Anticipating this issue will need to be part of the overall planning process for training enrolment scale-up.

While improvements in attrition over time do not have the same benefits on the long-term health workforce as improvements in training enrolment, reductions in attrition will reduce the magnitude of needed training enrolment increases that are required to meet targets--and, conversely, any increases in attrition over time will enlarge the magnitude of needed training enrolment increases that are required to meet targets. Two new government policies have the potential to reduce attrition. Zambia currently requires students in government-supported training institutions to work in the public sector workforce for a specified number of years after graduation under a bond. Efforts to enforce or even expand the time requirement of this policy should reduce attrition of newly hired health workers. Furthermore, the MOH is piloting retention schemes that provide monetary and working environment incentives to keep staff in the public sector, especially in rural areas [25]. Improving working conditions is a strategy that is likely to improve retention, as health staff who are operating under desirable working conditions and well equipped to do their job are more likely to have higher job satisfaction and remain in the public sector health system [35]. If successful and scaled up, these programs could provide strong complements to training enrolment increases, especially for doctors who have the highest attrition rates.

### Study limitations

This study has several limitations. Our intent in this analysis was to suggest training, hiring, and attrition conditions under which the MOH can reach its HRH target in the next ten years. Our conclusions should be interpreted with caution since we do not analyze the feasibility and costs that are associated with each intervention. A study was commissioned by the MOH subsequent to this analysis that examined the feasibility and costs of doubling training institution enrolment for all cadres by 2012 [27]. The findings of that cost study along with the results of our HRH strategy analysis allowed the MOH to understand the costs and benefits of investing in training institution enrolment, ultimately leading to the MOH's decision to increase training enrolment. More studies will be necessary to determine the feasibility and costs of improving hiring and retention conditions across all cadres in Zambia, with the ultimate goal of combining that costing information with this strategy analysis to understand the relative costs and benefits of all HRH policies.

This analysis assumes an unlimited demand for slots in training institutions. While anecdotal evidence suggests that there is currently a surplus of qualified applicants at the national training institutions, this may change if training institution enrolment were increased dramatically. Adequate preparation in primary and secondary education becomes even more crucial in the preparation of a good supply of eligible and qualified applicants. We also assume the MOH has an unlimited capacity to absorb newly-trained health workers. It is possible that the government will not be able to hire graduates of training institutions at current rates if the number of graduates grows. Historically, international monetary institutions have imposed limitations on the expansion of the public sector workforce in debtor countries [36]. Unforeseen changes to the labour market also have the potential to alter the government's ability to hire and retain graduates. Moreover, we caution that these estimates of workforce need are made at the national level, while staffing levels at the local level will vary, affecting access.

Our findings are based on a wide range of possible values for training institution enrolment, attrition, graduation rates, and public sector entry rates. Our projections assume the structure of the workforce will remain the same over time and does not incorporate potential changes in productivity such as those from task shifting and skill mix. We make a number of assumptions for the model parameters based on the best available data. As the population of Zambia continues to grow, it is likely that staffing targets will also increase given their rooting in population size; however, we do not update the staffing targets in our analysis to reflect this estimated growth but rather use the current MOH approved established targets for the health cadres. Furthermore, our analysis focused on a ten year horizon. If this time horizon is lengthened or shortened, our results would change.

Finally, our analysis suggests a rapid increase in the training enrolment in the next ten years, which could be followed by a large decrease after targets are reached. This would need to be taken into consideration down the road so as to avoid an equally rapid increase in attrition from the workforce as this large group of trainees that graduate in the next ten years retires or leaves the workforce.

## Conclusions

Closing the gap between the demand and supply of health workers in Zambia requires an increase in health training school enrolment. Supplemental interventions targeting attrition, graduation and public sector entry rates can help close the gap. HRH modelling provides a valuable tool to help policy makers examine how a range of policy options would impact the supply of HRH.

Following this analysis, the Government of Zambia called for an increase in current training enrolment by over 90% across all cadres. In May and June of 2008, the Government of Zambia and the Clinton Foundation assessed all 39 of Zambia's health training institutions to develop a full-cost estimate of the needs associated with meeting these expanded targets [27]. The Government is currently scaling-up enrolment through investments in infrastructure and faculty over the next five years.

## Abbreviations

AIDS: Acquired immune deficiency syndrome; CHAI: Clinton HIV/AIDS Initiative; HIV: Human immunodeficiency virus; HR: Human Resources; HRH: Human resources for health; MDG: Millennium Development Goal; MOH: Ministry of Health; WHO: World Health Organization;

## Competing interests

The authors declare that they have no competing interests.

## Authors' contributions

MK and KS conceived of the study, guided the analysis, and coordinated the collection of data. AT helped to develop the model, assisted in the collection of data, assisted in the sensitivity and what-if analyses, and helped to draft the manuscript. CS performed the sensitivity analysis and helped to draft the manuscript. EM helped to conceive the design of the study, coordinated the analysis, and helped to draft the manuscript. JL and ML performed the analysis of the training institutions and assisted with the collection of data. All authors contributed to the writing of and approve the content in the final manuscript.
